# Autoantibodies - enemies, and/or potential allies?

**DOI:** 10.3389/fimmu.2022.953726

**Published:** 2022-10-19

**Authors:** Hui Ma, Caroline Murphy, Christine E. Loscher, Richard O’Kennedy

**Affiliations:** ^1^ School of Biotechnology, Dublin City University, Dublin, Ireland; ^2^ Research, Development and Innovation, Qatar Foundation, Doha, Qatar; ^3^ Hamad Bin Khalifa University, Doha, Qatar

**Keywords:** autoantibody, diagnosis, treatment, autoimmune disease, cancer, COVID - 19, biomarker

## Abstract

Autoantibodies are well known as potentially highly harmful antibodies which attack the host *via* binding to self-antigens, thus causing severe associated diseases and symptoms (e.g. autoimmune diseases). However, detection of autoantibodies to a range of disease-associated antigens has enabled their successful usage as important tools in disease diagnosis, prognosis and treatment. There are several advantages of using such autoantibodies. These include the capacity to measure their presence very early in disease development, their stability, which is often much better than their related antigen, and the capacity to use an array of such autoantibodies for enhanced diagnostics and to better predict prognosis. They may also possess capacity for utilization in therapy, *in vivo*. In this review both the positive and negative aspects of autoantibodies are critically assessed, including their role in autoimmune diseases, cancers and the global pandemic caused by COVID-19. Important issues related to their detection are also highlighted.

## Introduction

It is well known that an antibody (Ab) produced by B cells helps the immune system to identify and neutralize non-self-antigens (e.g. antigens from bacteria, viruses, toxins and fungi etc.). However, sometimes the immune system fails to distinguish between self and non-self-antigens, leading to the generation of autoantibodies against self-antigens, or autoimmunity. This can cause autoimmune diseases, and, increasingly, autoimmunity has been found to be associated with a wide range of diseases, such as cancer, infectious disease (e.g. such as COVID-19), cardiovascular disease and neurodegenerative disease. However, autoantibodies are also found in healthy populations, albeit usually not in high levels and, for the most part, do not cause damage or attack the host.

Autoantibodies were first reported by Hargraves et al. (1) in lupus erythematosus (LE). LE cell factors, which could bind nuclear antigens, were eventually identified as autoantibodies. While it is widely reported that autoantibodies play a crucial role in the pathogenesis of various autoimmune diseases, they may mediate both systemic inflammation and tissue injury (2). The exact reasons for autoantibody generation in certain diseases (e.g. cancer) have still not been fully elucidated. However, there are several suggested theories for autoantibody generation in cancer. They may include a) tolerance defects and inflammation, b) altered antigen expression, c) changes in exposure or presentation of antigen, and d) cellular death mechanisms (3). Some autoantibody production is due to a combination of genetic and environmental factors (e.g. exposure to viruses, certain toxins and hazardous chemicals). In systemic lupus erythematosus (SLE), autoantibody generation is triggered by genetic abnormalities and environmentally induced defects in immune cells, mutations in regulatory components which are involved in cellular apoptosis and ineffectual cellular debris clearance (4).

Approximately 5% of the general population suffers from one or more autoimmune diseases (5). About 50 million Americans may have some form of an autoimmune disease and among them, over 75% are women. Autoimmune diseases cause significant deaths in young to middle-aged women (6, 7). In some cancers and other diseases autoantibodies are generated and these can be used as biomarkers in diagnosis. It is also suggested that some cancers may be promoted by certain autoantibodies (8, 9). Increasingly reports suggest that in COVID-19, especially severe COVID-19, new autoantibodies are generated (10). Moreover, these new autoantibodies directly cause harm, including blood clotting, blood vessel inflammation, and tissue damage. Autoantibodies may also play a role in long COVID symptoms (11).

Autoantibodies may provide both harmful and potentially beneficial effects. For example, in malaria, autoantibodies appear to be involved in aspects of pathogenesis, which then lead to various symptoms, such as cerebral malaria, anemia, acute kidney injury and respiratory distress syndrome. However, autoantibodies which are produced during malarial infection may also assist in host protection (12). Autoantibodies can also make useful contributions in disease diagnosis, prognosis and treatment. The longer half-lives of autoantibodies (when compared with their sometimes less stable antigens) *in vivo* (e.g. in blood and other bodily fluids) often makes autoantibody detection easier and more effective compared to measurement of their related antigens. The overall stability of autoantibodies (with half-lives of up to several weeks in blood circulation and over years when blood samples are stored at -20°C and even longer at -80°C) may be much higher than that of their associated antigen (sometimes with a half-life of a few hours or a few days in the blood circulation). For instance, the *in vivo* half-lives of IL-1b and IL-18 are 20 min and 16 h, respectively (13), while the average half-life of IgG autoantibody in circulation is about 3 weeks (14). Moreover, many autoantibodies may be detected well in advance of clinical manifestations of the disease, which enables earlier diagnosis and, thus, benefits the selection and application of effective treatments (15). In addition, the immune response against self-antigens amplifies the signal, which makes the autoantibody detection easier and earlier than antigen detection. Finally, autoantibody-based immunoassays (e.g. enzyme-linked immunosorbent assay (ELISA) and lateral flow) are very easy to be translated to clinical diagnosis platforms (16).

Since autoantibodies can be used as promising biomarkers for diagnosis/prognosis of various diseases (e.g. autoimmune diseases, cancer and cardiovascular disease) this may aid potential treatment using a more targeted approach (17, 18). It was also noted that patients with some autoimmune diseases have a lower cancer risk, which suggests that certain autoantibodies may contribute to the protection of the individual (19).

## Problems caused by autoantibodies

### Role of autoantibodies in autoimmune diseases

In autoimmune diseases, the immune system ‘mistakenly’ recognizes self-cells/tissues/organs as non-self, which leads to autoantibody production. Such autoantibodies then bind/attack self-cells/tissues/organs causing damage, inflammation and/or organ dysfunction. However, some autoantibodies do not cause injury directly (e.g. Graves’ disease is directly caused by thyroid-stimulating autoantibodies), but they are thought to be part of an overall complicated immune response that causes inflammation and damage. Various roles of autoantibodies in relation to the activation of autoimmune diseases are detailed below.

There are approximately 100 identified autoimmune diseases (20). Nine of the most common and/or harmful autoantibody-associated autoimmune diseases are Type 1 diabetes (T1D), rheumatoid arthritis (RA), multiple sclerosis (MS), systemic lupus erythematosus (SLE), Graves’ disease (GD), psoriasis, inflammatory bowel disease (IBD), Sjögren’s syndrome (SS) and celiac disease (CD).

It was reported that 9.5% of the world’s population was affected by T1D, in which the immune system attacks insulin-producing cells in the pancreas (21). Insulin, produced by the pancreas plays a crucial role in blood sugar regulation. High blood sugar caused by T1D leads to damage of blood vessels and organs (e.g. heart, kidneys, eyes, and nerves). Many different harmful autoantibodies are generated including autoantibodies against glutamic acid decarboxylase (GAD), an enzyme which aids pancreatic function. Such anti-GAD autoantibodies promote T1D. They do this by directing the immune system to kill the insulin-producing pancreatic cells. Moreover, autoantibodies against islet cell cytoplasmic insulinoma-associated antigen-2, as well as insulin were usually found in T1D patients. These autoantibodies also are involved in T1D development (22).

In rheumatoid arthritis (RA) the immune system attacks the joints resulting in their damage and destruction and, eventually, disability occurs. Approximately 0.5-1% of adults worldwide suffer from RA (23). Autoantibodies are also important biomarkers for RA, and among these are rheumatoid factor (autoantibody against the fragment-crystallizable (Fc) region of IgG) and antibodies against post-translationally modified proteins involving citrullination (ACPA) and carbamylation (anti-CarP antibodies). Immune complexes in the joint may be formed by these autoantibodies, which cause swelling, redness, stiffness and warmth (24, 25).

Multiple sclerosis (MS) is a chronic autoimmune disease, where the protective coating (known as myelin sheath) that surrounds nerve cells in the central nervous system is damaged, and this is associated with characteristic inflammatory lesions and demyelination. Mobility limitations are a key feature, while other typical symptoms are weakness, numbness, balance dysfunction and trouble with walking. Over 2.8 million people are estimated to live with MS worldwide (26) and about 50% of these people will require the use of a walking aid within 15 to 25 years following diagnosis with MS (27). Kuerten et al. (28) demonstrated that B cells and autoantibodies play crucial roles in MS pathogenesis, also, broadly increased anti-myelin autoantibody levels were detected in the plasma of MS patients. Identification of specific pathogenic autoantibodies in MS and their target antigens remains a significant challenge.

Systemic lupus erythematosus (SLE) is well known to cause skin rash, however, it is a chronic autoimmune disease which affects many organs, including the joints, kidneys, brain, and heart. Other common symptoms of SLE are joint pain/swelling, fatigue, and fever.

It was reported that the prevalence of SLE in 2018 in the US was 0.073% (29). Autoantibody production in SLE is thought to be triggered by a complex interaction of genetics, the environment, and hormones, leading to harmful self-attack and inhibition of immune regulation (30). Leptin, an adipocytokine, plays a crucial role in the development and maintenance of proinflammatory immune responses and SLE was found to be promoted by autoantibodies increased by leptin (31).

Graves’ disease (GD) is caused by thyroid-stimulating autoantibodies (TSAbs) that activate the thyrotropin receptor on the thyroid cell membrane, leading to the over-production of thyroid hormones. GD affects approximately 2-3% of the world’s population (32). While thyroid hormones play a crucial role in control of metabolism, high levels hyper-stimulate the body’s activities, resulting in nervousness, a fast heartbeat, heat intolerance, and weight loss. Bulging eyes, an associated symptom of GD, was found in circa 30% of GD patients (33).

Psoriasis is an autoimmune condition where T cells mistakenly attack the host’s skin cells. It is a common skin disease affecting approximately 2–3% of the population globally (34). The occurrence of psoriasis in children varied from 0% in Taiwan to 2.1% in Italy, whilst in adults it ranged from 0.91% in the USA to 8.5% in Norway (35). In psoriasis, skin cells grow too quickly. This leads to build up of extra cells and this causes inflamed red patches. Over 30% of psoriasis patients also develop psoriatic arthritis, a form of inflammatory arthritis that can cause pain, swelling and sometimes damage to the joints. Four autoantigens involved in psoriasis have been reported. They are cathelicidin LL-37, melanocytic ADAMTSL5, lipid antigen PLA2G4D and keratin 17. Autoantibodies against LL-37 and ADAMTSL5 have been reported to play a potential role in pathogenesis of psoriatic arthritis (36).

Inflammatory bowel disease (IBD) is an immune-mediated inflammatory disease, which causes inflammation in the lining of the intestinal wall. There are two types of IBD, namely Crohn’s disease and ulcerative colitis (UC) (37). Crohn’s disease can produce inflammation in any part of the gastrointestinal tract, from the mouth to the anus, whereas ulcerative colitis affects only the lining of the large intestine (colon) and rectum. It is reported that autoantibodies may promote the pathological phenotype by activating M1 monocytes in the animal model where NOD/ScidIL2Rγnull mice were reconstituted with PBMC from ulcerative colitis donors (NSG-UC), and also in patients with ulcerative colitis (38). Antinuclear autoantibodies (ANA, the antibodies that attack contents in the cell nucleus) may represent a factor that enhances the propensity to the development of ulcerative colitis (39).

Sjögren’s syndrome is an autoimmune disorder where the immune system attacks the glands providing lubrication to the eyes and mouth and leads to dry eyes and dry mouth. It also affects the joints and skin. About 0.2-4% of the world’s population are affected (40). There are various autoantibodies associated with Sjögren’s syndrome. These include anti-salivary protein 1 (SP1), anti-carbonic anhydrase II and IV, anti-parotid secretory protein (PSP), anti-Ro (SS-A) and anti-La (SS-B), rheumatoid factor, and ANA (41, 42).

The pathogenic role of these autoantibodies in the development of Sjögren’s syndrome remains to be elucidated. However, Kim et al. (43) reported a pathologic role of primary Sjögren’s syndrome autoantibodies associated with down-regulation of the major histocompatibility complex I (MHC I) molecule with muscarinic type 3 receptor (M3R) through internalization. It was also found that autoantibodies against Ro and La cause apoptosis in the A-253 cell line. Moreover, anti-carbonic anhydrase II autoantibodies have been detected in approximately 13 to 21% of Sjögren’s syndrome patients and are believed to have a pathogenic role in renal tubular acidosis, a common extra-glandular manifestation of primary Sjögren’s syndrome (44).

Celiac disease (CD), an autoimmune enteropathy, is triggered by dietary gluten in genetically susceptible individuals. The pooled global prevalence of CD was 1.4%, based on serologic test results published from January 1991 through March 2016 (45). In CD, the immune system attacks the small intestine with gluten in it, which leads to inflammation, diarrhea and abdominal pain. While the autoantibodies in CD do not trigger the disease directly, they have pathogenic potential. It is reported that CD autoantibodies induced ataxia *in vivo*, and, moreover, induce epithelial proliferation and neuronal apoptosis *in vitro* (46). CD can be easily identified by the presence of autoantibodies against a self-antigen, tissue transglutaminase (tTG). The specificity of anti-tTG autoantibodies (IgA and IgM in IgA-deficient subjects) test can achieve 99% for CD patients (47).

### Autoantibodies which promote the progression of cancer

Autoantibodies are produced in the early stage of cancer by the humoral immune response, which is activated by abnormal expression of tumor-associated antigens (TAAs) (48). The presence of autoantibodies is well established as early-stage biomarkers in cancer diagnosis. However, some autoantibodies are believed to contribute to cancer progression and resistance to cancer therapy, while some contribute to cancer suppression (19, 49, 50). Lin et al. (51) reported two autoantibodies (antibody 93 and 641) in breast cancer patients which play a role in cancer progression. Antibody 93 stimulated tumor growth, while antibody 641 inhibited tumor cell growth. The role of autoantibodies in promoting and inhibiting cancer is poorly defined. Andreu et al. (52) reported that autoantibodies promote chronic-inflammation-induced tumorigenesis. They found that through interaction with activating Fc gamma receptors, they may control several crucial functions of leukocytes in neoplastic tissue. It was also noted that stromal accumulation of autoantibodies in premalignant skin appears to promote neoplastic progression and subsequent carcinoma development.

It is reported that among patients with autoimmune diseases, that the risk of certain cancers is increased significantly. For instance, hematological, vulvar, thyroid, pancreatic, lung and hepatic cancers occur more frequently in SLE patients (53). Anti-double stranded DNA (anti-dsDNA) autoantibodies were found in about 30% of SLE patients but were less than 1% in healthy individuals. These anti-dsDNA autoantibodies, which attack and damage DNA, cause increased release of intracellular contents (e.g. DNA) from dying cells, which may then lead to further inflammation and autoantibody production, and, thus form a destructive cycle. Anti-dsDNA autoantibodies could increase the cancer risk directly among SLE patients through DNA damage or inflammation. (8, 9).

Some non-B cell-produced antibodies were found to influence tumor progression. For example, antibodies or ‘antibody-like’ molecules produced by malignant epithelial cells of various tumors (e.g. breast tumor, colon tumor, lung tumor and liver tumor) were able to aid the growth and survival of tumor cells *in vitro* and *in vivo* (54). This leads to the intriguing question as to whether or not these are autoantibodies and preliminary evidence suggests some are, as they target self-antigens (e.g. HEp2 cell antigens) (49, 55). However, the situation is still complex. Findings seem to indicate that antibodies expressed and secreted by various cancer cells (e.g. colorectal cancer, epithelial cancer, prostate cancer) enable cancer cell proliferation but suppress cancer cell apoptosis (56–58). Tumor-derived antibodies have been found to aid tumor development and progression in the following aspects i.e. tumor cell growth and proliferation, tumor cell migration, invasion, and metastasis; tumor immune escape and other biological functions (e.g. immunity regulation, promotion of drug resistance, involvement in cancer-associated diabetes, influence of tumor-associated thrombosis, mediation of CSC potential, regulation of cell morphogenesis, cell cycle process, fatty acid biosynthetic process, protein biosynthesis, and antimicrobial activity) (55). Xu et al. (57) found out that after IgG_1_ knockdown, colony formation, survival, cell cycle progression, migration and invasion of LNCaP (lymph node carcinoma of the prostate) cells decreased significantly. Furthermore, reduction of proliferation [assessed *via* the proliferation marker, proliferating cell nuclear antigen (PCNA)], and increasing of numbers of apoptotic cells (detected using the apoptotic marker, caspase-3) were observed after IgG_1_ silencing.

### Autoantibodies could drive severe and long COVID-19

Several publications have suggested a link between autoantibody generation and severe/long COVID-19 (10, 59–62). It is reported that 52% of 172 people hospitalized with COVID-19 had autoantibodies against phospholipids, which contribute to the control of blood clotting, which is one of the severe COVID-19-associated symptoms (63). Therefore, scientists concluded that these anti-phospholipid autoantibodies are potentially pathogenic. It was demonstrated that autoantibodies neutralizing high concentrations of type I interferons (IFNs) were found in 9.5% of patients admitted to the ICU for COVID-19 pneumonia in a hospital in Barcelona (64). Troya et al. (65) also reported in a hospital in Madrid, anti-type I IFN autoantibodies were found in over 10% of COVID-19 patients at severe/critical COVID-19 stages. Moreover, Chauvineau-Grenier et al. (66) reported that the presence of anti-type I IFN autoantibodies was associated with higher risk of mortality, as these autoantibodies were found in 21% of patients who died from COVID-19 pneumonia in a hospital in France. Therefore, the presence of these anti-type I IFNs autoantibodies was recommended for testing as soon as possible after COVID-19 diagnosis, as they may indicate the possibility of life-threatening issues. Inhibition of annexin A2 leads to systemic thrombosis, cell death, and non-cardiogenic pulmonary edema. Zuniga et al. (67) reported increased anti-annexin A2 autoantibodies among hospitalized COVID-19 patients and these autoantibody levels were associated with and may predict mortality levels. Anti-annexin A2 autoantibodies can be included in testing to predict severe COVID-19.

Cytokines are crucial in the immunopathology of infections caused by viruses, including COVID-19. They are well known to assist the immune and inflammation responses *via* controlling the growth and activity of blood cells and cells of the immune system. Increased levels of a wide range of cytokines [e.g., interferon (IFN)-α, IFN-ϵ, IL-1β, IL-2, IL-6, IL-7, IL-8, IL-10, IL-17, IL-22, macrophage colony-stimulating factor (M-CSF), granulocyte colony-stimulating factor (G-CSF), granulocyte-macrophage colony stimulating factor (GM-CSF), 10 kD interferon-gamma-induced protein (IP-10), monocyte chemoattractant protein-1 (MCP-1), macrophage inflammatory protein 1-α (MIP 1-α) and TNFα] as well as anti-cytokine autoantibodies have been identified in hospitalized/severe COVID-19 patients (68). Feng et al. (69) reported that more than 60% of hospitalized COVID-19 patients have one or more autoantibodies that recognize cytokines. Interestingly enough, these anti-cytokine autoantibodies are also highly prevalent (over 50%) in non-COVID-19 infections patients in ICU. Moreover, Chang et al. (10) stated that various autoantibodies (including anti-cytokine autoantibodies and autoantibodies against some intracellular antigens) found in COVID-19 patients are also associated with connective tissue diseases (e.g. systemic sclerosis, myositis, and overlap syndromes). It was found that autoantibodies against IFN-γ, GM-CSF, IL-6 and TH-17 contribute to or are closely related to infection susceptibility (70). Bastard et al. (71) reported that autoantibodies against type I IFNs neutralize their corresponding type I IFNs to block COVID-19 infection both *in vitro* and *in vivo*.

But one key question is, do pre-existing autoantibodies cause severe COVID-19 or does COVID-19 trigger the production of new autoantibodies which then cause severe COVID-19 symptoms, or both? Some scientists suggested that pre-existing autoantibodies against type I IFNs are predictive of critical COVID-19 pneumonia (64). However, more and more publications showed that new autoantibodies developed during and after COVID-19 (especially severe COVID-19) cause serious problems (10, 72). Wang et al. (73) found autoantibodies which attacked B cells, and some that attacked interferon in COVID-19 patients. They also suggested the possibility that COVID-19 triggers the body to generate new autoantibodies which attack self-tissues. Such autoantibodies were found against proteins in patients’ blood vessels, hearts and brains. These new autoantibodies can do harm to individuals by causing blood clotting, blood vessel inflammation, tissue/organ/nerve damage, and attack the immune system, resulting in impaired ability to fight infection (74).

It is highly possible that both pre-existing and new autoantibodies generated during/after COVID-19 infection play a crucial role in severe COVID-19 individuals. Chang et al. (10) screened blood (serum and plasma) samples from 147 hospitalized COVID-19 patients, and then concluded that 52% of the patients with severe COVID-19 had at least one type of pre-existing autoantibody in their blood, while in healthy controls, only 15% had these autoantibodies. They also found that about 20% of hospitalized COVID-19 patients did not have any autoantibodies when they were first admitted but developed them during their illness. This evidence suggests that while pre-existing autoantibodies do correlate with severe COVID-19 it also leads to the development of new-onset IgG autoantibodies. These new autoantibodies, which break tolerance to self, were also found to correlate positively with the severity of COVID-19 (10).

Liu et al. (72) demonstrated that COVID-19 triggers the development of autoantibodies directly. These new autoantibodies were against both structural proteins similar to COVID-19, but also to proteins which are dissimilar to COVID-19 proteins. Moreover, they found sex-specific patterns of autoantibody reactivity, which last up to 6 months following associated symptomatology. Namely, males carry the risk of diverse autoimmune activation following symptomatic COVID-19, while females carry the risk for a distinct profile of autoimmune activation following asymptomatic COVID-19.

Many scientists believed that autoantibodies may play a role in long COVID symptoms, as these autoantibodies can remain for over 6 months, or much longer, after the original COVID-19 virus disappeared (11). Therefore, relevant autoantibody presence could be tested at an early stage following diagnosis of COVID-19, to predict which patients are at high risk or, for long COVID, with a view to more specific and effective treatment. (64).

## Exploiting the benefit of autoantibodies

### Autoantibody value in the detection and treatment of autoimmune and other disorders

Autoantibodies are widely used in the diagnosis and prognosis of autoimmune diseases (**Table 1**). For some autoimmune diseases, the diagnosis can be easily achieved through the observation of symptoms and detection of autoantibodies. For example, Graves’ disease and Hashimoto’s thyroiditis can be easily diagnosed and monitored by anti-thyroid autoantibody levels (95). Celiac disease can mainly be diagnosed and monitored by anti-tTG and DGP autoantibody tests (112). Moreover, some autoantibodies may be detected many years before the onset of autoimmune disease. It was reported that over 88% of SLE patients showed at least one SLE autoantibody-positive test (e.g. aANA, antiphospholipid, anti-Ro, anti-La, anti-Sm, anti-nuclear ribonucleoprotein and anti-double-stranded DNA autoantibodies) before the diagnosis of SLE (up to 9.4 years earlier; mean, 3.3 years) (114).

**Table 1:** Summary of autoantibody applications in various autoimmune disease.

Autoimmune disease type	Autoantibody targets for diagnosis and prognosis	Autoantibody targets for treatment
Type 1 diabetes (T1D)	Insulin; cytoplasmic proteins in beta cells, glutamic acid decarboxylase (GAD-65); protein tyrosine phosphatase (IA-2A); zinc transporter 8 (ZnT8); Pdx1 and Reg1A, cytokine CCL3, Rab GDP dissociation inhibitor beta (GDIb) (75)	CD3, CD20, CD2, interleukin (IL)-1β, IL-1R, IFNα, IFNγ, IL-12, IL-21, IL-17A, IL-25, CD4 and CD8α, CD127, IL-7Rα, IL-2, CD127, IL-7Rα (76)
Rheumatoid arthritis (RA)	Rheumatoid factor (RF), citrullinated antibodies (ACPA), carbamylated protein (anti-CarP), peptidyl arginine deiminase-4 (PAD-4), glucose-6-phosphate isomerase (anti-GPI), Type II collagen (CII), Heterogeneous nuclear ribonucleoprotein A2, RA33, malondialdehyde (MDA), malondialdehyde-acetaldehyde (MAA), CCP2 (25, 77)	TNFα, integrin alpha-9 (α9), IL-2, IL-10, IL-6R, CD20, CD80/86 (78, 79)
Multiple sclerosis (MS)	Potassium channel KIR4.1 (80), anti-α-D-Glcp-(1→4)-α-D-Glcp (GAGA4) IgM (81), myelin oligodendrocyte glycoprotein (MOG), myelin basic protein (MBP), KIR4.1, Neurofilaments-Heavy chain (NF-H), Chitinase-3-Like-1 precursor, miR-19a, miR-21, miR-22, miR-142-3p, miR-146a, miR-146b, miR-155, miR-210, and miR-326 (82)	Integrin α, α4β1-integrin, CD52, CD20 (83–85);
Systemic lupus erythematosus (SLE)	C1q, panel of Erythrocyte-bound C4d (E-C4d), B-cell-bound C4d (B-C4d), nuclear contents, and mutated citrullinated vimentin (MCV), panel of antibodies against dsDNA, nuclear contents, MCV, E-C4d, and B-C4d (86); Serum Complement 3 (C3), Complement 4 (C4), Nucleosome, Erythrocyte Sedimentation Rate (ESR), C-Reactive Protein (CRP), Sm, C1q, C1q (87); extractable nuclear antigens (anti-ENA), B lymphocyte stimulator (BLyS), TNF-like weak inducer of apoptosis (TWEAK) (88), myc-associated zinc finger protein (MAZ) (89), TRIM21 (90)	B-lymphocyte stimulator (BLyS) (91); Thyroid peroxidase (TPO), BLyS, CD 20, CD22, CD19, Cereblon Modulator (CC-220), CD40, IL-12/23, IL-10, IL-6, IFNα, Interferon-γ (IFNγ), IFNα Kinoid (IFNα-K) (92) CD38 (93) a proliferation-inducing ligand (APRIL) (94)
Graves’ disease (GD)	Thyroglobulin (TG), thyroid peroxidase (TPO), and thyroid-stimulating hormone receptor (TSHR) (95)	CD20 (96), TSHR/IGF-IR receptor complex, IL-6, TNF-α (97)
Psoriasis	Cyclic Citrullinated Peptide, Rheumatoid factor (RF), nuclear contents (98)	TNF-α, (99) IL-12, -23, and -17 (100) CD6 (101), CD4 (102)
Inflammatory bowel disease (IBD)	Neutrophil cytoplasmic, Exocrine Pancreas, *Saccharomyces cerevisiae*, glycan, outer-membrane porin C, Cbir1, I2, *Mycobacterium avium* subspecies paratuberculosis, *Caenorhabditis elegans*, cocktail multiple antigenic peptide, tailless complex polypeptide (TCP), granulocyte macrophage colony-stimulating factor (103, 104)	TNF-α, IL-12, IL-23, α4 integrin, α4β7 integrin (105–107)
Sjögren’s syndrome (SS)	Nuclear contents, Sjögren’s syndrome type, rheumatoid factor, Panel of murine parotid tissue proteins, including parotid secretory protein, carbonic anhydrase 6, and salivary protein-1 (108), carbonic anhydrase 6 (CA6) (109)	CD-20 (110), IFN-α (111)
Celiac disease (CD)	Tissue Transglutaminase (tTG), Deamidated gliadin peptide, endomysium (112)	IL-15 (113)

Nevertheless, for the autoimmune diseases which involve systemic autoantibodies against various organs or systems (e.g. rheumatoid arthritis, systemic lupus erythematosus, scleroderma, and dermatomyositis), the diagnosis is much harder. For instance, in order to diagnose systemic lupus erythematosus, in addition to symptom assessments, physical examination and X-rays, levels of various autoantibodies should be determined against a panel of targets, namely, anti-erythrocyte-bound C4d (E-C4d), anti-B-cell-bound C4d (B-C4d), ANA, and anti-mutated citrullinated vimentin (MCV); or, against a panel including anti-dsDNA, ANA, anti-MCV, anti-E-C4d, and anti-B-C4d (86).

Profiling the autoantibodies presented in serum is commonly used for the diagnosis of diseases, including various autoimmune diseases and cancers. Protein array technology enables the identification of novel panels of autoantibody biomarkers through the screening of the humoral immune response against thousands of proteins (115). For example, thousands of recombinant proteins may be expressed, purified, and then spotted on microarrays or chips to enable easy screening of test samples (e.g. serum) (116–118). Moreover, the potential autoantibodies can be screened and identified using tiny amounts of samples [e.g. autoantibodies to over 10,000 human antigens can be investigated in one experiment using only 50 µL serum sample by applying Engine protein arrays (Engine – a biomarker company in Germany)]. Antigens thus identified can then be used as potential novel probes for disease diagnosis, stage, progression, response to therapy, as well as treatment (119). For example, B-lymphocyte stimulator (BLyS) has been found to increase significantly in SLE patients, and autoantibodies against BLyS have been successfully applied in SLE treatment (120). An anti-BLyS human monoclonal antibody, Belimumab, was approved by the US FDA, and is still proven to be safe and effective in SLE therapy (121).

Scientists have shown great interest in anti-idiotypic (anti-ID) use. Here the antibody against the binding site of the autoantibody (Ab1) is known as anti-ID antibody (Ab2). Autoantibodies offer a potential target for anti-ID antibody prevention/treatment for autoimmune disease. The antigen-binding regions of anti-ID antibodies (Ab2) that are specific for autoantibody (Ab1) can structurally mimic/resemble that of the target antigens. Thus, the Ab2 antigen-binding domain can potentially represent an exact mirror image of the initial targeted antigen of Ab1. Moreover, Ab2 may display a similar functional activity with the original antigen (122). Such Ab2s have been validated for potential application as a surrogate for the original antigen in vaccine studies (123).

There are several advantages of using anti-ID antibodies for immunotherapy. Firstly, anti-ID antibodies enable the inhibition of specific autoantibody responses while the rest of the immune system remains unaffected, thus, avoiding potential side effects. Secondly, anti-ID antibodies trigger a memory response (through T-helper memory cell generation) promoting longer-lasting immunity and preventing relapses. Finally, it is relatively safe to use anti-ID antibodies *in vivo*, since anti-ID antibodies naturally occurred in the body and the immune response caused by anti-ID antibodies should be similar with that caused by the original antigens, which are mimicked by anti-ID antibodies (124). For example, high levels of anti-dsDNA autoantibodies can be detected years before the onset of SLE, and these harmful autoantibody levels are associated with the severity of SLE. Anti-dsDNA autoantibodies are often correlated with continuing inflammation and kidney damage (125). Lee et al. (126) reported that high levels of anti-dsDNA antibody were successfully neutralized and decreased through the binding of anti-ID antibodies, which then lead to apoptosis of anti-dsDNA antibody-producing cells. Therefore, there is potential to employ anti-ID antibodies for prevention (e.g. vaccine) and treatment purposes.

**Table 1** summarizes the targets for autoantibodies which have been used clinically or have high potential (as proved at research level) in diagnosis, prognosis and therapy of autoimmune diseases. It is very interesting to notice that for diagnosis and prognosis of different types of autoimmune disease, the autoantibody targets vary significantly for disease-specificity, while for treatment, one target can be used in several different types of autoimmune disease. For example, anti-cluster of Differentiation 20 (CD20) autoantibody showed therapeutic effect in Type 1 diabetes, rheumatoid arthritis, multiple sclerosis, systemic lupus erythematosus, Graves’ disease and Sjögren’s syndrome. Anti-tumor necrosis factor (TNF) α autoantibody is effective in the treatment of rheumatoid arthritis, Graves’ disease, psoriasis and inflammatory bowel disease. Anti-IL-12 autoantibody can be useful for the therapy of Type 1 diabetes, systemic lupus erythematosus, psoriasis and inflammatory bowel disease. This indicates that a panel of autoantibodies may be able to provide effective treatment for various autoimmune diseases/conditions.

### The role of autoantibody in cancer diagnosis, prognosis and tumor inhibition/treatment

Cancer requires early detection and effective treatment, while early diagnosis is usually essential for effective therapy. Tumor-associated autoantibodies are popular candidates for both early detection and treatment of cancer, as increasing numbers of autoantibodies against tumor specific antigens have been reported and their detection exploited for research and clinical analysis (127–129).

Tumor-associated autoantibodies are antibodies produced as an immune response against various tumor-associated autoantigens (i.e. over expressed antigens, mutated or post-translationally modified proteins). Various tumor-associated autoantibodies have been identified in virtually all types of cancers. However, there are some problems associated with autoantibody detection. This includes issues due to their very low levels which may be undetectable (130). Autoantibodies may start to be produced before disease symptoms are manifested but their detection may be difficult due to their low concentrations. Some autoantibodies may be very good potential biomarkers (sensitive and specific), but there is the possibility that their corresponding target antigens are still not identified, either because they are unknown or have not been linked to specific diseases (131). There may also be an issue for detection of autoantibodies that have very low antigen binding affinities (132).

Kijanka et al. (133) demonstrated that by screening high-density protein arrays, colorectal cancer-special antibody profiles (e.g. autoantibodies against p53, HMGB1, TRIM28, TCF3, LASS5 and ZNF346) can be identified for colorectal cancer diagnosis in symptomatic patients. Fitzgerald et al. (134) further described a novel ELISA assay which showed high predictive value for the presence of colorectal cancer, through the detection of IgM and IgG autoantibody immune responses in human serum. This novel blood-based test has potential for enhanced patient uptake, as such a blood-based test is generally more acceptable than a fecal-based test.

Some autoantibodies have potentially good diagnostic capacity, but some are also applicable for use in prognosis. It is reported that the serum autoantibodies against GAGE7, MAGEA1, PGP9.5, CAGE and p53 could be used for lung cancer diagnosis, while autoantibodies to PGP9.5 particularly correlate with poor prognosis for lung cancer patients (135). Some autoantibodies were associated with good prognosis, while others indicated bad prognosis. Denkert et al. (136) found that high levels of autoantibodies against tumor-infiltrating lymphocytes (TILs) and p53 were associated with better prognosis in HER2-positive breast cancer patients, while autoantibodies against MSH2, EZR, PGK1, VCL and ANXA2 were associated with poorer pancreatic cancer patient outcomes (137). Autoantibodies against CDC25B also indicated poor prognosis in advanced esophageal squamous cell carcinoma (ESCC) patients (138). In some circumstances, an autoantibody can be used in both diagnosis and treatment (139) with panels of autoantibodies being more useful. For example, an autoantibody against human epidermal growth factor receptor 2 (HER2) can be used for diagnosis for HER2-positive breast cancer. Moreover, as HER2 promotes cancer cell growth, anti-HER2 autoantibodies may also provide potentially effective anti-cancer outcomes (140). For prostate cancer, an anti-prostate-specific antigen (PSA) autoantibody could serve as a diagnostic biomarker (since PSA is a FDA-approved prostate cancer diagnosis biomarker, although it has clearly established limitations, but is routinely used in clinics), and may mediate anti-cancer effects (141). The value of anti-PSA antibodies was shown by Sinha et al. (142) who successfully used anti-PSA IgG as a selective delivery agent for conjugated chemotherapeutic drugs to PSA-producing neoplastic prostate cells in nude mice, without causing cytotoxic effects on mouse organs.

Early diagnosis improves cancer outcomes significantly, but, is also challenging and demanding. Wang et al. (143) identified an anti-ALDH1B1 autoantibody which may have potential for early detection of colorectal cancer. Anti-TOPO48 autoantibody was reported to be a potentially useful biomarker for early diagnosis and prognosis of ESCC (144). Autoantibodies against aberrantly glycosylated MUC1 in early-stage breast cancer are believed to predict a better prognosis (145). Detection of a panel of autoantibodies against seven various targets (p53, GAGE7, PGP9.5, CAGE, MAGEA1, SOX2 and GBU4-5) was suggested to have significant clinical value for early diagnosis of lung cancer (146). However, autoantibody panels are generally proven to be more effective and accurate than the use of a single autoantibody in cancer diagnosis (147). O’Reilly et al. (148) reported that a panel of zinc finger proteins, including ZNF346, ZNF638, ZNF700 and ZNF768, are suitable for use as capture antigens in a blood-based autoantibody biomarker assay for colorectal cancer. Jiang et al. (48) successfully developed a panel of seven autoantibodies (reactive with: TP53, NPM1, FGFR2, PIK3CA, GNA11, HIST1H3B, and TSC1) for effective early detection of lung cancer, as well as providing novel targets for lung cancer immunotherapy.

In relation to the determination of autoantibodies, IgG, IgM and IgA have been demonstrated to have differential discriminatory abilities (134, 149). While protein targets are predominant, changes in glycation, citrullination or phosphorylation are also potentially significant. The linkage of autoantibody profiles to genomic findings and other sets of clinical data, with associated analysis using complex computational approaches, should be capable of providing greater insights and diagnostic capacity and risk analysis of diseases including cancer and autoimmunity (150).

Increasing numbers of studies have shown that some autoantibodies play a crucial role in cancer inhibition. Autoantibodies could inhibit tumors *via* surveillance mechanisms thereby controlling prolonged survival for various cancers, for instance, melanoma, esophago-gastric, breast gastric, colon, lung, pancreatic and tongue cancers (19). It is reported that the generation of autoantibodies specific to tumor antigens is derived from the migration, differentiation, and maturation of TIL-B (tumor infiltrating B-cells) in tumor-associated tertiary lymphoid structures. Thus tumor associated autoantibodies, which are believed to be indicative of more significant immunological reactivity, will induce functional anti-tumor humoral immunity and promote immune surveillance for cancer cells (151). Evidence suggests that certain pre-existing tumor associated autoantibodies (e.g. NY-ESO-1, XAGE1, and SIX2) are associated with clinical benefit in anti-PD-1 treatment for non-small-cell lung cancer (152–154). Karagiannis et al. (155) observed that impairment of autoantibody IgG1-mediated tumoricidal functions, generated poor clinical outcomes in melanoma.

The autoantibodies or autoantibody-derived antibodies involved in cancer therapy function in four ways. Firstly, autoantibodies induce tumor cell death directly, which includes blockade of growth factor receptor signaling, as well as ligand binding blockage that induces tumor cell apoptosis. Secondly, autoantibodies induce tumor cell death indirectly by engaging components from the host immune system, which include antibody-dependent cellular cytotoxicity, complement-dependent cytotoxicity, and antibody-dependent cellular phagocytosis (156). Thirdly, neutralization of harmful tumor-specific antigens and/or overexpressed tumor-associated antigens may occur. Finally, delivery of chemotherapy or radiotherapy specifically to cancer cells, but not to healthy cells and tissues, can be mediated with less side effects (157). There are now many potential autoantibodies for use in diagnosis and therapy of various cancers. For instance, there are around 100 autoantibodies with possible utility for lung cancer diagnosis and therapy (48, 146, 158, 159). **Table 2** summarizes autoantibody targets which have been approved by the EU and US FDA for cancer diagnostics and therapeutics.

**Table 2:** Summary of EU and/or US FDA-approved autoantibody targets for cancer diagnosis and of autoantibodies/autoantibody-derived antibodies for cancer treatment .

Cancer Type	Autoantibody targets for diagnosis	Treatment autoantibodies or autoantibody-derived antibody drug conjugates (ADC) (Antibody name/target/type)
Breast cancer	HER2/neu, CA27-29, and CA15-3 (Mucin-1 [MUC1])	Margetuximab/HER2/Chimeric IgG; Atezolizumab/PD-L1/Humanized IgG1; Ado-trastuzumab emtansine/HER2/Humanized IgG ADC; Pertuzumab/HER2/Humanized IgG1; Trastuzumab emtansine/HER2/Humanized IgG1; [fam]-trastuzumab deruxtecan/HER2/ Humanized IgG1 ADC; Margetuximab/HER2/Chimeric IgG1; Sacituzumab govitecan/TROP2/Humanized IgG1 ADC.
Lung cancer		Atezolizumab/PD-L1/Humanized IgG1; Bevacizumab/VEGF/Humanized IgG1; Necitumumab/EGFR/recombinant human IgG1; Nivolumab/PD-1/Human IgG4; Pembrolizumab/PD-1/Humanized IgG4.
Bladder cancer	Nuclear Mitotic Apparatus protein (NuMA, NMP22)	Atezolizumab/PD-L1/Humanized IgG1; Durvalumab/PD-L1/Human IgG1; Enfortumab/vedotin Nectin-4/human IgG1.
Colorectal cancer	carcinoembryonic antigen (CEA)	Bevacizumab/VEGF/Humanized IgG1; Cetuximab/EGFR/Chimeric IgG1; Edrecolomab/EpCAM/Murine IgG2a; Panitumumab/EGFR/Human IgG2; Nivolumab/PD-1/Human IgG4; Ramucirumab/VEGFR2/Human IgG1.
Renal /kidney cancer		Bevacizumab/VEGF/Humanized IgG1; Ipilimumab/CTLA-4/Human IgG1; Nivolumab/PD-1/Human IgG4.
Ovarian cancer	CA125 (MUC16), ROMA (HE4+CA-125), OVA1 (multiple proteins), HE4	Bevacizumab/VEGF/Humanized IgG1.
Multiple Myeloma		Belantamab mafodotin/BCMA/Humanized IgG1 ADC; Daratumumab/CD38/Human IgG1; Elotuzumab/SLAMF7/Humanized IgG1; Isatuximab/CD38/Chimeric IgG1.
Melanoma		Ipilimumab/CTLA-4/Human IgG1; Nivolumab/PD-1/Human IgG4; Pembrolizumab/PD-1/Humanized IgG4; Tebentafusp/gp100 CD3/Bispecific immunoconjugate (TCR-scFv).
Lymphoma		Loncastuximab tesirine/CD19/Humanized IgG1 ADC; Tafasitamab/CD19/Humanized IgG1 ; Mogamulizumab/(T cell) CCR4/Humanized IgG1; Rituximab/(B cell) CD20/Chimeric IgG1; Brentuximab vedotin/CD30/Chimeric IgG1 ADC; Polatuzumab vedotin/CD79B/Humanized IgG1 ADC; Ibritumomab tiuxetan/CD20/Murine IgG1; Iodine (I131) tositumomab/CD20/Murine IgG2a; Pembrolizumab/PD-1/Humanized IgG4.
Leukemia		Moxetumomab pasudotox/CD22/Murine IgG1 dsFv-immunotoxin; Obinutuzumab/CD20/Humanized IgG1; Ofatumumab/CD20/Human IgG1; Glycoengineered Blinatumomab/CD19, CD3/Murine bispecific tandem scFv; Alemtuzumab/CD52/Humanized IgG1; Inotuzumab ozogamicin/CD22/recombinant humanised IgG4 ADC; Gemtuzumab ozogamicin/CD33/Humanized IgG4 ADC.
Sarcoma		Olaratumab/PDGFRα/Human IgG1.
Gastric cancer		Ramucirumab/VEGFR2/Human IgG1.
Cervical cancer		Tisotumab vedotin/Tissue factor/Human IgG1 ADC; Pembrolizumab/PD-1/Humanized IgG4.
Pancreatic cancer	CA19-9	LYT-200/galectin-9/human IgG4.
Prostate cancer	PSA	Evaluating Panitumumab/(ABX-EGF) EGFr/human IgG2.
General cancer type	Carcino-embryonic antigen	

This table is derived from a combination of reports (156, 157, 160).

The advantages and principles of using the anti-ID antibodies in treatment were stated previously. Racotumomab (Vaxira) is the first approved (approved only in Cuba and Argentina) anti-ID antibody therapeutic vaccine. Racotumomab, which is well tolerated by patients, has successfully increased the overall survival rate of Non-Small Cell Lung Cancer patients in recurrent or advanced stages (161).

### How could autoantibody utilization aid the treatment of COVID-19?

Certain autoantibodies (e.g. anti-type I IFNs autoantibody) could drive severe and long COVID-19. These ‘harmful’ autoantibodies should be determined at an early stage following diagnosis of COVID-19 infection to predict the severity and possible long-term effects of infection, thus, hopefully enabling more effective therapy. Anti-cytokine autoantibodies (e.g., antibodies against IFNα, IFNϵ, IL-6, IL-22, GM-CSF and TNFα) may also provide a potential target for COVID-19 treatment. Troya et al. (65) analyzed clinical data from COVID-19 patients receiving subcutaneous IFN-beta-1b treatment from March to May 2020, at the Infanta Leonor University Hospital in Madrid, Spain. However, no improved clinical outcomes were observed. It was suggested that IFN-beta treatment was given too late, after two weeks of symptoms. Therefore, an earlier, ambulatory IFN-beta treatment appears to be required (162).

Cytokines play an important role in protection of the host against bacterial and viral (including COVID-19) infections. However, an over-activated immune response may cause an acute inflammatory reaction called a ‘cytokine storm’ (acute overproduction and uncontrolled release of pro-inflammatory cytokines), leading to multiple organ dysfunction. This is quite common (ca 21%) in COVID-19-infected pneumonia patients (163–165). Therefore, autoantibodies could be employed for COVID-19 treatment. The therapeutic functions of monoclonal neutralization antibodies against IL-6 and GM-CSF have been reported. Temesgen et al. (166) successfully used an anti-human GM-CSF monoclonal antibody for the treatment of patients with severe COVID-19 pneumonia, which proved to be safe and effective, with improved clinical outcomes, as well as a reduced cytokine storm. Moreover, an anti-IL-6 monoclonal antibody decreased IL-6 levels, which lead to the reduction of the inflammatory process in COVID-19 patients with severe respiratory disease. Therefore, there is high potential to use anti-IL-6 neutralization antibody for prevention of a cytokine storm and death caused by it (167). Similarly, anti-ID antibodies, which showed significant value for autoimmune disease treatment could be used in the same way.

Autoantibody-triggered autoimmune responses are often associated with severe and long COVID-19. Therefore, anti-ID antibodies of autoantibody targets may also have potential in COVID-19 treatment (122, 168, 169).

## Conclusions and future trends

Autoantibodies have various roles and can be exploited as enemies, as well as friends, capable of doing harm and good. The levels and stability of autoantibodies can cause challenges (e.g. in autoimmune disorders and long COVID-19), but also enable potentially better and more reliable diagnosis.

Overall, in order to take full advantage of autoantibodies, and avoid/limit their negative aspects, more research is required. Luckily, more and more mature and advanced technologies will aid research on autoantibodies, for instance, protein arrays and use of anti-ID antibodies (119, 169).

The use of protein arrays for autoantibody detection offers advantages including high multiplexing capacity, availability of multiple detection systems, well-established quality control procedures, small sample volume requirements, high sensitivity, good dynamic ranges and rapid generation of results. Challenges include the need to detect autoantibodies at highly variable concentrations, issues with effective immobilization of proteins/antigens depending on their characteristics, epitope availability and stability and the need to identify appropriate sets of targets with the required sensitivity and specificity (170). The availability of artificial intelligence (AI) and other approaches for processing results from multiple analytical determinations from many patient cohorts and controls should also provide enhanced discrimination for diagnosis and follow-up. Linking autoantibody determination with genomics analysis should provide opportunities for precision health for greatly improved patient welfare, but the associated analysis may be complex (171).

## Author contributions

HM and RO’K contributed to the conception and design of the work; HM drafted the work; CM, CL and RO’K revised it critically for important intellectual content and provided approval for publication of the content. All authors contributed to the article and approved the submitted version.

## Funding

This work was supported by Science Foundation Ireland, grant number 14/IA/2646.

## Conflict of interest

The authors declare that the research was conducted in the absence of any commercial or financial relationships that could be construed as a potential conflict of interest.

## Publisher’s note

All claims expressed in this article are solely those of the authors and do not necessarily represent those of their affiliated organizations, or those of the publisher, the editors and the reviewers. Any product that may be evaluated in this article, or claim that may be made by its manufacturer, is not guaranteed or endorsed by the publisher.
